# Integrated Pharmacological Analysis on the Mechanism of Fuyou Formula in Treating Precocious Puberty

**DOI:** 10.3389/fphar.2021.649732

**Published:** 2021-05-07

**Authors:** Chunyan Guo, Ning Sun, Kaili Hu, Guoliang Bai, Meng Zhang, Qian Wang, Qian Ding, Jing Liu, Xiaoling Wang, Libo Zhao

**Affiliations:** ^1^ Department of Pharmacy, National Center for Children Health, Beijing Children’s Hospital, Capital Medical University, Beijing, China; ^2^ School of Traditional Chinese Medicine Department, Beijing University of Chinese Medicine, Beijing, China; ^3^ Department of Traditional Chinese medicine, National Center for Children Health, Beijing Children’s Hospital, Capital Medical University, Beijing, China

**Keywords:** integrated pharmacology, precocious puberty, Chinese medicine, protein-protein interaction network, Fuyou formula

## Abstract

Fu-you formula (FY), a Traditional Chinese Medicine (TCM) formula composed of 12 herbs, as an in-hospital preparation, has been used treat to precocious puberty (PP) for decades. However, the lack of phytochemical characterization and mechanism of FY remains the main limitation for its spreading. In this study, we analyze the components and mechanisms of FY in PP, based on the integrated pharmacology. Investigated main constituents, targets, pathways of FY by using an integrative pharmacology, and recognized main constituents by HPLC-MS/MS. Then, observed the levels of Follicle-stimulating hormone (FSH), luteinizing hormone (LH), and estrogen (E_2_) in danazol-induced PP in Sprague–Dawley (SD) rats. Lastly, retrospective study analyzed the clinical data of 575 patients who were diagnosed PP, treated by the FY, and followed-up in our hospital from 2014–2020. The result that total of 116 important candidate targets were selected based on pharmacological analysis. Selected the top 10 values key targets such as the estrogen receptor alpha (ESR1) and insulin-like growth factor 1 (IGF1), were localized and the related gene functions were determined. Gene functions were associated with biological regulation, a cellular process, or signaling pathway, such as the Estrogen signaling pathway, MAPK signaling pathway and PI3K-Akt signaling pathway. By recognizing the five compounds related to the ESR1 and IGF1, which are Quercetin, kaempferol, Luteolin, Apigenin, and Emodin. The results of the molecular docking study further showed that the flavonoids had a strong binding affinity for ESR1 and IGF1 after docking into the crystal structure. The results showed that the FY could effectively reduce E_2_, LH, and FSH levels in SD rats. Furthermore, the results of the retrospective analysis of medical records showed that the FY could remarkably reduce E_2_ levels in girls with PP.

## Introduction

Precocious puberty (PP) is a common endocrine disorder among children. It occurs before the age of eight years in girls and before the age of 9 years in boys. In recent years, the annual incidence of this condition has been on the rise, and the incidence among girls is 5–10 times that among boys (Chinese Society of Pediatric Endocrinology and Metabolism (CSPEM), 2015). At present, early initiation of the gonadal axis is believed to be the cause of PP. Therefore, modern medicine holds the view that the administration of a gonadotropin releasing hormone antagonist (GnRHa) in the treatment of PP is the most effective method. However, clinical results show that the long-term use of a GnRHa has inhibitory effects on growth and the thyroid in children. In addition, some children require simultaneous treatment with growth hormone or even thyroxine. Clinical studies on the FY as a treatment for girls with PP at our hospital have shown that it can control the early symptoms, and effectively reduce estrogen levels and bone age (Liu et al., 2009; Pan et al., 2019). At present, the literature comprises mostly clinical reports and observations of curative effects. However, in-depth research on the effective components, key targets, and mechanisms of action of the FY are still lacking. Integrative pharmacology could enhance our comprehension and facilitate the prediction of potential targets, pathways, and effects, which might provide clues for the design of subsequent research studies. In the present study, we used an integrative pharmacological approach to understand the systemic, organ-related, and molecular effects of the FY. The components and mechanisms of the FY in the treatment of PP were preliminarily analyzed and explored. The TCM integrated pharmacology platform was used and a TCM-component-network target-disease multi-level network was considered as the underlying framework.

## Materials and Methods

### Materials and Reagents

Danazol was obtained from the A&D Technology Corporation (Beijing, China). Leuprorelin acetate microspheres for injection were purchased from Livzon (Zhuhai, China). FSH, LH, and E_2_ ELISA kits were obtained from Cloud-Clone Corp (Wuhan, China). The TCM standards Quercetin (serial number: 100081–201610, purity: 99.90%), Luteolin (serial number: 111520–202006, purity: 94.40%), kaempferol (serial number: 110861–202013, purity: 93.20%) and Emodin (serial number: 110756–201913, purity: 96.0%) were purchased from the National Institutes for Food and Drug Control (Beijing, China). Apigenin (serial number: B20981–20 mg, purity: 98.00%) was purchased from the Shanghai Yuanye Bio-Technology Co., Ltd. (Shanghai, China).

### Plant Materials and Fu-you Formula Preparation

The FY was an in-hospital preparation (Approval number: Z20053679). It comprised a mixture of *Prunella vulgaris* L. (Xiakucao); *Carapax Trionycis* (Cubiejia); *Gentiana scabra* Bunge (Longdan); *Chrysanthemum morifolium* (Ramat.) Hemsl (Juhua); *Lycium chinense* Mill (Digupi); *Alisma plantago-aquatica* L (Zexie); *Scrophularia ningpoensis* Hemsl (Xuanshen); *Paeonia suffruticosa Andrews* (Mudanpi); *Rehmannia glutinosa* (Gaertn.) DC (Shengdihuang); *Hordeum vulgare* L (Maiya); *Concha oetreae* (Muli); *Thalluslaminariae* (Kunbu) (1.5:1:0.6:0.6:1:1:1.5:0.6:1.2:2:3:1). All herbs were purchased from the Beijing Bencao Fangyuan Pharmaceutical Group Co. Ltd. and the FY was prepared by the Preparation Center of the Beijing Children's Hospital (Lot number: 20201202).

### Construction of the Compound-Target and Disease-Target Database

To identify the corresponding targets of the 12 active ingredients of the FY, several approaches combining chemometric methods, information integration, and data mining were implemented. First, all active compounds were submitted to the TCM-IP platform (http://www.tcmip.cn/TCMIP/index.php/Home/Index/index.html) Xu et al. (2019), as well as the TCMSP (http://tcmspw.com/tcmsp.php) Ru et al. (2014), and TCMID (http://119.3.41.228:8000/tcmid/search/) Huang et al. (2018) to mine compound-target interactions. The biological targets of the active ingredients were obtained from the STITCH (http://stitch.embl.de/) Szklarczyk et al. (2016); SwissTarget (http://www.swisstargetprediction.ch/) Gfeller et al. (2014); CTD (http://tcmspw.com/index.php) Yan et al. (2019); and SymMap (https://www.symmap.org/) Wu et al. (2019) databases. Known therapeutic targets for PP were obtained from the DrugBank (http://www.drugbank.ca/) Wishart et al. (2018); Online Mendelian Inheritance in Man (OMIM) (http://www.omim.org) Hamosh et al. (2005); and DisGeNET (https://www.disgenet.org/home/) Janet et al. (2020) databases.

### Protein-Protein Interaction Network Construction

The protein-protein interaction (PPI) data were imported from the STRING (https://string-db.org/cgi/input.pl?sessionId=rEkaDRgfV0vC&input_page_show_search=on) PPI databases. An interactive network for the candidate drug targets and known PP-related targets of the FY was constructed based on their interaction data and was visualized using the Cytoscape software (Shannon et al., 2003). Interactions between the targets of the traditional Chinese medicine components of the FY and the targets related to PP were determined. Furthermore, the gene interaction network of the Chinese medicine components of the FY and PP was established. The degree centrality (DC) equal to two times the median value, was applied as the core for selection of the network nodes (hubs node). Thus, the median of node connectivity, closeness centrality, and betweenness centrality were the key values that determined the selection of nodes. Nodes that met three values simultaneously were selected as the candidate key targets of the FY in the treatment of PP.

### Gene Oncology Enrichment and Pathway Analysis

We performed gene ontology (GO) analysis of the non-repetitive putative targets of the FY using the database for Annotation, Visualization, and Integrated Discovery (DAVID) to gain insights into their involvement in two different categories namely, biological process and molecular function (Sherman and Lempicki, 2009). Tissue enrichment analysis was performed using the FunRich software (http://www.funrich.org) (Pathan et al., 2015). We then performed Kyoto Encyclopedia of Genes and Genomes (KEGG) signaling pathway enrichment analysis of the candidate targets of the FY after topological analysis. A *P*-value < 0.05 was considered significant, and the enriched GO terms were identified using the hypergeometric test. A bubble chart was plotted using the OmicShare tools, a free online platform for data analysis (www.omicshare.com/tools).

### Chemical Components Analysis

Characterization of main chemical components in FY was assayed by HPLC-MS/MS (AB SCIEX QTRAP 5500). Chromatographic separation was performed on a Hypersil Gold C18 column (150 × 2.1 mm, 5 μm) (Thermo Scientific), with column temperature set at 40°C. The mobile phase was solution A, 2 mM ammonium acetate in water containing 0.4‰ formic acid, and solution B, methanol. Gradient elution program was: 0–1.5 min, 60–10% A; 1.5–3.5 min, 10% A; 3.5–3.51 min, 10–60% A; 3.51–6.0 min, 60% A. The flow rate of mobile phase was 0.4 ml/min. The mass spectrometer was operated in negative ion mode with a needle potential of -4,500 V; the source temperature was set at 500°C. Nitrogen was used as the sheath gas and auxiliary gas at pressures of 50 and 40 psi. Multiple reactions monitoring (MRM) mode was used to identify the five compounds by monitoring their transitions from the molecular ions to product ions. The proper amounts of standard substance were weighed and dissolved in methanol-water (1:1, v/v) to prepare standard solutions at 1 μg/ml. Meanwhile, 10 μL of FY was mixed with 1 ml of methanol-water (1:1, v/v) by vortexing for 10 min, then centrifuge for 15 min at 15,000 rpm. The supernatant fluid was used as sample solution. The chromatograms of standard solution and sample solution were used to compounds matching.

### *In Silico* Molecular Docking

*In silico* molecular docking studies of bio-active peptides or chemical drug molecules that exert their action by binding with specific receptors provides evidence on binding conformation, pattern and affinity. To identify the binding ability of active constituents with PP related targets, the crystal structures of ESR1 (PDB code: 6VIG) and IGF-1 (PDB code: 1IMX) were obtained from RCSB Protein Data Bank (http://www.rcsb.org/), and three main compounds structure of Quercetin, Apigenin and Luteolin were obtained from PubChem (https://pubchem.ncbi.nlm.nih.gov/) to establish molecular docking model with Discovery Studio 4.5. The CDOCKER module of Dock Ligands in Discovery Studio 4.5 was used to do the docking. The kinetic method was used to randomly search the small molecule conformation, and then the simulated annealing method was used to optimize each conformation in the receptor active site region, so as to make the docking results more accurate.

### Animals

At postnatal day (PND) 3, female Sprague-Dawley rats and their mothers were obtained from SPF Biotechnology Co., Ltd. (Beijing, license no: SYXK (Beijing) 2016–0038). The rats were housed in the laboratory animal room and maintained at 24 ± 2°C, with 42 ± 5% humidity on a 12-h light/dark cycle (lights on from 07:30 to 19:30) in a specific-pathogen-free animal room. The animals were supplied food and water *ad libitum* and acclimated for three days before the start of the experiments. All animal experiments were performed in strict compliance with Chinese guidelines, including the standards for Laboratory Animals (GB14925–2001), and the Guideline on the Humane Treatment of Laboratory Animals (MOST 2006a). All animal procedures were approved by the Beijing Administration Office for Laboratory Animals.

### Animal Grouping and Drug Administration

The animals were randomly divided into four groups: the control group, model group, positive control (leuprorelin) group, and FY group. At PND 5, the rats in the three experimental groups were given a single subcutaneous injection of 300 µg/25 µL danazol (ethylene glycol:ethanol = 1:1, v/v). The rats in the control group were given a subcutaneous injection of 25 µL of glycol/ethanol (Morishita et al., 1993; Ju et al., 2019). The rats in the positive control (leuprorelin) group were subcutaneously injected with 100 μg/kg leuprorelin. The rats in the FY group were given a solution formulated with dry ointment powder, by intragastric administration every day. The rats in the control and model groups were given the same amount of normal saline. The rats that exhibited vaginal opening were sacrificed at diestrus after a complete estrous cycle. The remaining rats were sacrificed at the same time point. All rats were anesthetized with an intraperitoneal injection of 2% pentobarbital sodium. Blood samples were collected from the abdominal aorta before sacrifice. Blood serum was separated by centrifugation (3,500 rpm, 20 min, 4°C) and preserved at −80°C for further analysis of serum hormone levels.

Drug dosage: The Fy dose was calculated according to the clinical dosage administered to 6-year-old girls. According to the following formula:

$$d_{\text{B}}=d_{\text{A}}\text{dA}\times R_{\text{B}}/R_{\text{A}}\times {\left(W_{\text{A}}/W_{\text{B}}\right)}^{1/3}$$

with *d*_B_ representing the animal/human body weight dose, *d*_A_ representing the known human/animal body weight dose, *W*_A_ and *W*_B_ representing known human and animal weights, respectively, and *R*_A_ and *R*_B_ representing known human/animal body shape coefficients, respectively. Every two days, the animals were weight, and the dose was recalculated.

### Serum Hormone Level Detection

After anesthesia, blood was collected from the abdominal aorta, and the serum was centrifuged at 4°C and stored at −20°C until further analysis. The serum concentrations of FSH, LH, and E_2_ were measured using ELISA kits, according to the manufacturers’ instructions. The ELISA kits, which employ a competitive inhibition enzyme immunoassay technique, were purchased from Cloud-Clone Corp (Wuhan, China).

### Retrospective Analysis of Cases

Children with PP, treated with the FY at the outpatient department of our hospital from 2014 to 2020 were also evaluated. The inclusion criteria were as follows: 1) continuous use of the FY for 1 year; and 2) evaluation of sex hormone levels every 6 months. The exclusion criteria were as follows: 1) the presence of other endocrine diseases; 2) the presence of ovarian cysts. This retrospective study was approved by the Medical Ethics Committee of Beijing Children’s Hospital, Capital Medical University.

### Statistical Analysis

All results were presented as the mean ± SD. Differences were analyzed using one-way ANOVA. The data were further analyzed and plotted using the SPSS 19.0 software (IBM SPSS Software, New York, United States). Differences were considered statistically significant at *p* < 0.05.

## Results

### Formula Analysis of Fu-you Formula

*Prunella vulgaris* L.and *Carapax Trionycis* act on the liver and relieves congestion, nourishes yin and clears heat; *Gentiana scabra* Bunge, *Chrysanthemum morifolium* (Ramat.) Hemsl, *Lycium chinense* Mill, *Alisma plantago-aquatica* L, *Scrophularia ningpoensis* Hemsl.*, Paeonia suffruticosa* Andrews, *Rehmannia glutinosa* (Gaertn.) DC, which clear heat and removes dampness, nourishes yin, and cools the blood; *Hordeum vulgare L*, *Concha oetreae* and *Thalluslaminariae* act on the liver and relieves congestion, used as an adjuvant. All herbs combined act on the liver, clear congestion, nourish yin, and clear heat, can reduce the size of nodules in the breast, eliminate vaginal secretions, and dissipate scrofula, and reduce sputum production. The TCM composition are listed in Table 1.

**TABLE 1:** Composition of the FY.

Chinese name	Scientific name	Family	Lot No	Place of origin	Parts of plant used
Xia Ku Cao	*Prunella vulgaris* L.	Lamiaceae	20201010	Jiangsu, China	Dried erial parts
Cu Bie Jia	*Carapax Trionycis*	Trionyxsinensis Wiegmann	20201018	Hubei, China	Carapace
Long Dan	*Gentiana scabra* Bunge	Gentianaceae	20200927	Yunan, China	Dried roots and rhizomes
Ju Hua	*Chrysanthemum morifolium* (Ramat.) Hemsl	Compositae	20201027	Anhui, China	Capitulum
Di Gu Pi	*Lycium chinense* Mill	Solanaceae	20201105	Hebei, China	Dried root bark
Ze Xie	*Alisma plantago-aquatica* L	Alismataceae	20201126	Fujian, China	Dried tuber
Xuan Shen	*Scrophularia ningpoensis* Hemsl	Scrophulariaceae	20201019	Zhejiang, China	Dried root tuber
Mu Dan Pi	*Paeonia suffruticosa* Andrews	Paeoniaceae	20201123	Anhui, China	Dried root bark
Sheng Di Huang	*Rehmannia glutinosa* (Gaertn.) DC	Plantaginaceae	20201104	Henan, China	Dried root tuber
Mai Ya	*Hordeum vulgare* L	Triticum	20201030	Hebei, China	Dried ripe fruit
Mu Li	*Concha oetreae*	Ostrea	20200917	Guangdong, China	Shell
Kun Bu	*Thalluslaminariae*	Laminaria	20200922	Fujian, China	Dried lobes

### Chemical Composition and Prediction Target Analysis

Four hundred and thirteen chemical components were detected from 12 TCM in the FY. Furthermore, 37,468 predicted drug targets were obtained. The predicted target information and the characterization of each herb were derived from data on the TCM-component-targets, as shown in Table 2. Analysis of the common targets among the predicted targets, yielded a total of 4,741 predicted targets, among the 12 TCM. No common intersection targets were detected among the 12 TCM. 97 targets were detected between the two key herbs, which each had common targets with other herbs, as shown in Table 3.

**TABLE 2:** Basic data on the components and targets of traditional Chinese medicines.

TCM	Number of components	Number of targets
*Prunella vulgaris* L.	39	10,189
*Carapax Trionycis*	16	374
*Gentiana scabra* Bunge	45	1289
*Chrysanthemum morifolium* (Ramat.) Hemsl	101	11,384
*Lycium chinense* Mill	22	1358
*Alisma plantago-aquatica* L	26	795
*Scrophularia ningpoensis* Hemsl	25	564
*Paeonia suffruticosa* Andrews	36	9072
*Rehmannia glutinosa* (Gaertn.) DC	49	1777
*Hordeum vulgare* L	32	496
*Concha oetreae*	10	56
*Thalluslaminariae*	12	114

**TABLE 3:** Number of common targets.

TCM	*Prunella vulgaris* L.	*Carapax Trionycis*	*Gentiana scabra* Bunge	*Chrysanthemum morifolium* (Ramat.) Hemsl	*Lycium chinense* Mill	*Alisma plantago-aquatica* L	*Scrophularia ningpoensis* Hemsl	*Paeonia suffruticosa* Andrews	*Rehmannia glutinosa* (Gaertn.) DC	*Hordeum vulgare* L	*Concha oetreae*	*Thalluslaminariae*
*Prunella vulgaris* L.	—	97	358	3975	417	262	168	3991	293	181	25	61
*Carapax Trionycis*	97	—	48	95	58	37	30	88	43	41	7	16

### Construction and Analysis of Compound-Target Network of Fu-you Formula

**FIGURE 1:**
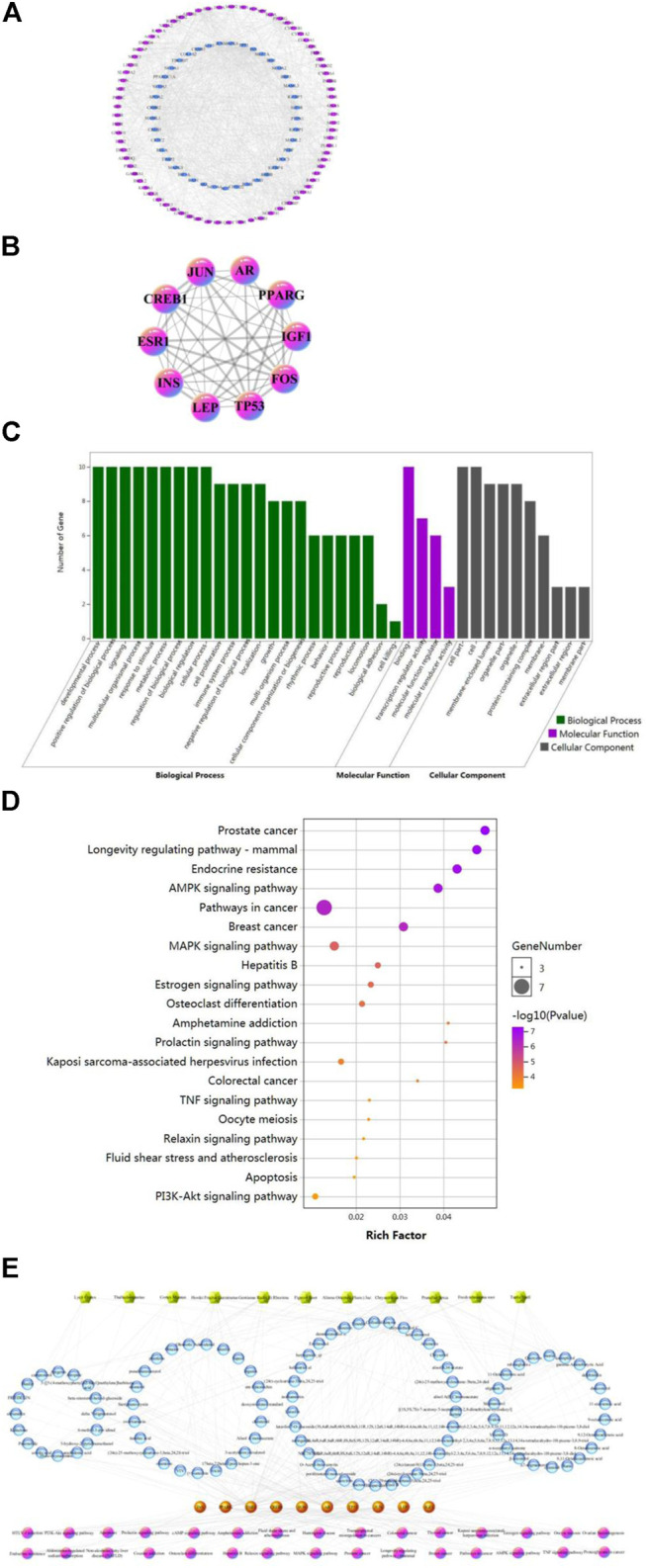
Compound-target interaction network and preliminary gene ontology (GO) analysis of drug targets. **(A)** Compound-key target network of the FY. **(B)** Compound-hub target network of the FY. **(C)** GO analysis of drug targets classified into three categories: biological process, molecular function, and cellular component. **(D)** Kyoto Encyclopedia of Genes and Genomes (KEGG) pathway analysis of core targets of the FY in the treatment of PP. **(E)** Chinese medicinal materials-core component-key target-main pathways.

After removing redundant targets, 4,741 targets obtained from 12 herbs intersected with 166 disease targets to obtain 79 mapped-genes. We then explored the predicted therapeutic targets of the FY, using multiple online databases as previously described. A network of potential targets of the compounds in the FY was then constructed using the Cytoscape software, as shown in Figure 1A. Based on the 1,224 core nodes obtained, 116 key candidate targets for the treatment of PP girls were screened out, 75 were direct targets and 41 were predicted targets. The degree values were determined for the top 10 hub genes, two of the most important targets are ESR1 and IGF1. After further analysis, there were 96 chemical components acting on 10 hub genes in the formula, and 42 chemical components acting on ESR1 and IGF1, which the five components most widely distributed in medicinal materials were Luteolin, Quercetin, Apigenin, Kaempferol and Emodin. The interaction relationships between targets were determined and a network map of the hub targets for the treatment of PP in girls was constructed (Figure 1B).

### Gene Oncology and Kyoto Encyclopedia of Genes and Genomes Enrichment Analysis of Hub Targets for the Fu-you Formula in Precocious Puberty

Based on the results of GO and KEGG pathway analyses, the enriched pathways were determined. A total of 2,462 hub genes were identified based on the GO analysis, which were associated with the target genes or proteins of cells (cellular component), molecular functions (function), or biological processes (in process). Gene function information is presented in Figure 1C. A total of 133 KEGG pathways were enriched, which were associated with key candidate targets. The top 20 pathways were sorted by *P*-value, as shown in Figure 1D.

### Construction and Analysis of the Multi-Layer Network Correlation Diagram of Traditional Chinese Medicine-Core Component-Hub Target-Main Pathways in the Treatment of PP With the Fu-you Formula

The KEGG pathways of the top 30 key candidate targets, based on their *P*-values were selected to construct the multi-level network association diagram of the “traditional Chinese medicine-core component-key target-main pathways” for the FY in the treatment of PP in girls (Figure 1E). The *P-*values were used to sort 30 KEGG pathways, which included 10 hub genes.

### Phytochemical Characterization of Fu-you Formula

**FIGURE 2:**
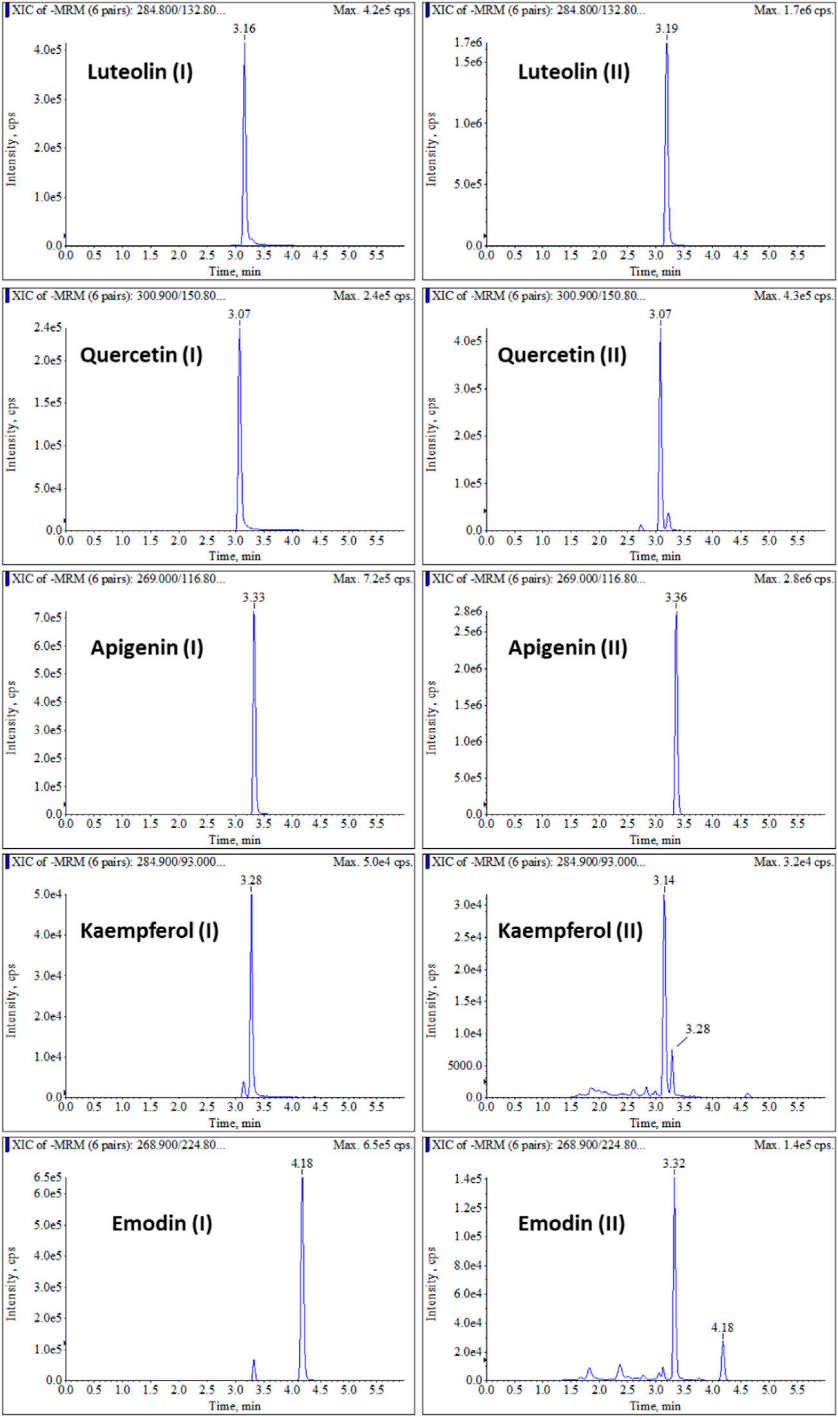
Chromatograms of HPLC-MS/MS of FY. [(I) Standard solution; (II) Sample solution of FY; Retention time of Kaempferol and Emodin were 3.28 and 4.18 min, respectively].

To identify the main constituents of FY, we analyzed the FY using HPLC-MS/MS. Five compounds were recognized from FY as shown in Table 4. The standard solution and the sample solution were analyzed by the HPLC-MS/MS method upper to identify the five constituents of FY. Accroding to the chromatograms (Figure 2), the consistent chromatographic peaks of the five compounds could be recognized in standard solution and sample solution, including of Luteolin, Quercetin, Apigenin, Kaempferol and Emodin. Therefore, it is convincing that FY contains these five constituents.

**TABLE 4:** MRM transitions for identify of the target compounds.

Analyte	Precursorion (m/z)	Production (m/z)	DP	EP	CE	CXP
Luteolin	284.8	132.8	−150	−2	−40	−40
Quercetin	300.9	150.8	−120	−2	−27	−40
Apigenin	269.0	116.8	−150	−2	−40	−40
Kaempferol	284.9	93.0	−180	−2	−40	−40
Emodin	268.9	224.8	−150	−2	−36	−40

### Molecular Docking

**FIGURE 3:**
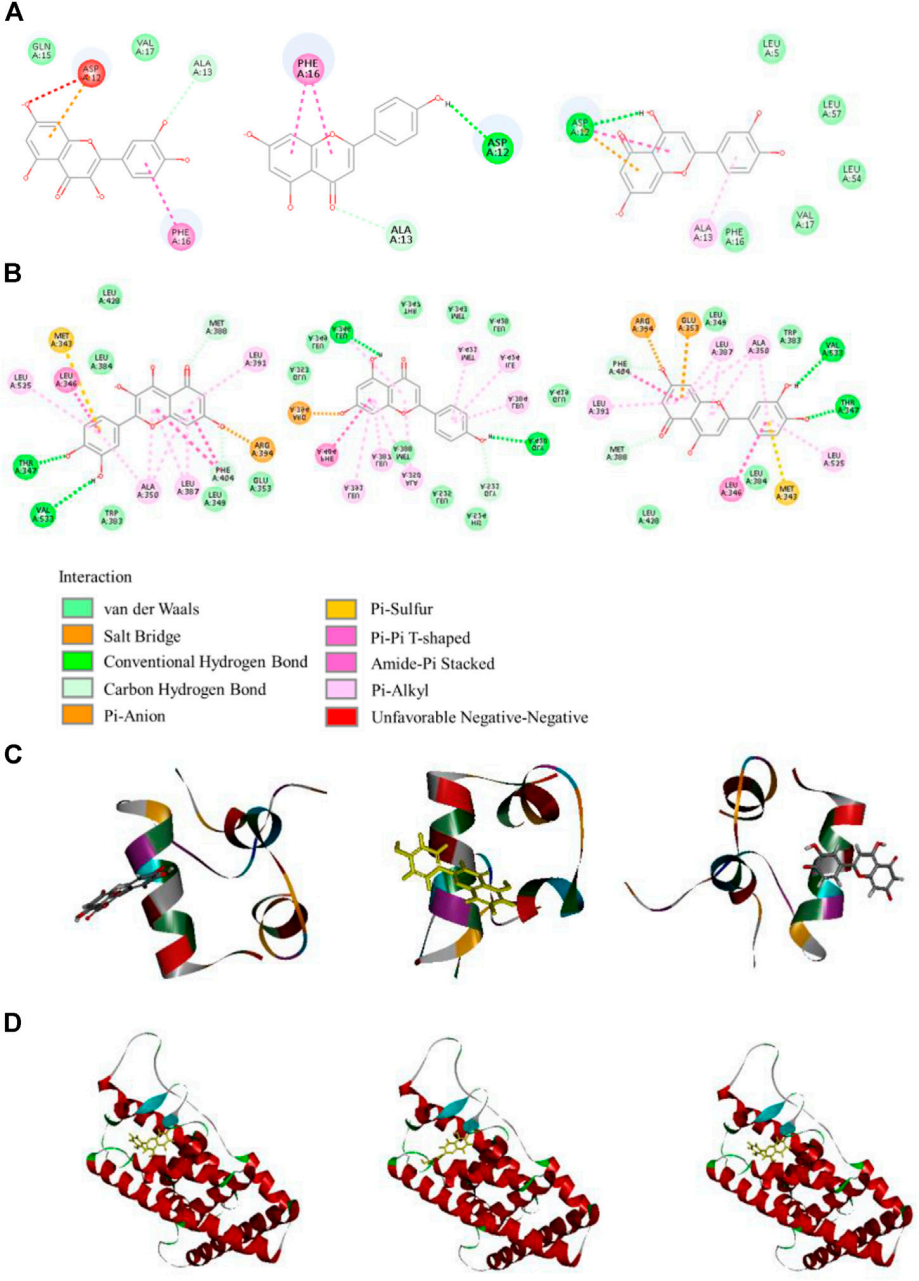
**(A)** Active binding sites of compounds with IGF1. **(B)** Active binding sites of compounds with ESR1. **(C)** Molecular docking patterns of compounds and IGF1. **(D)** Molecular docking patterns of compounds and ESR1.

To further validate the potential targets possessing good affinity to the ingredients, molecular docking was performed for 3 high content ingredients with the 2 high relevance degree proteins. The docking results of the 3 flavonoids with the target proteins ESR1 and IGF1 are shown in Table 5 and Figure 3. As shown in the results, all the active compounds have favorable binding energy (<0 Kcal/moL) with their relative potential target proteins, and 3 flavonoids interact with ESR1 more stronger, which adds chips to the reliability of the virtual screening results.

**TABLE 5:** Molecular docking results of target and active compounds.

Active compounds	Binding energy (Kcal/moL)	
	IGF1	ESR1
Quercetin	−9.81275	−41.9021
Apigenin	−16.8225	−43.4102
Luteolin	−13.2934	−40.1986

### Serum Test Results

**FIGURE 4:**
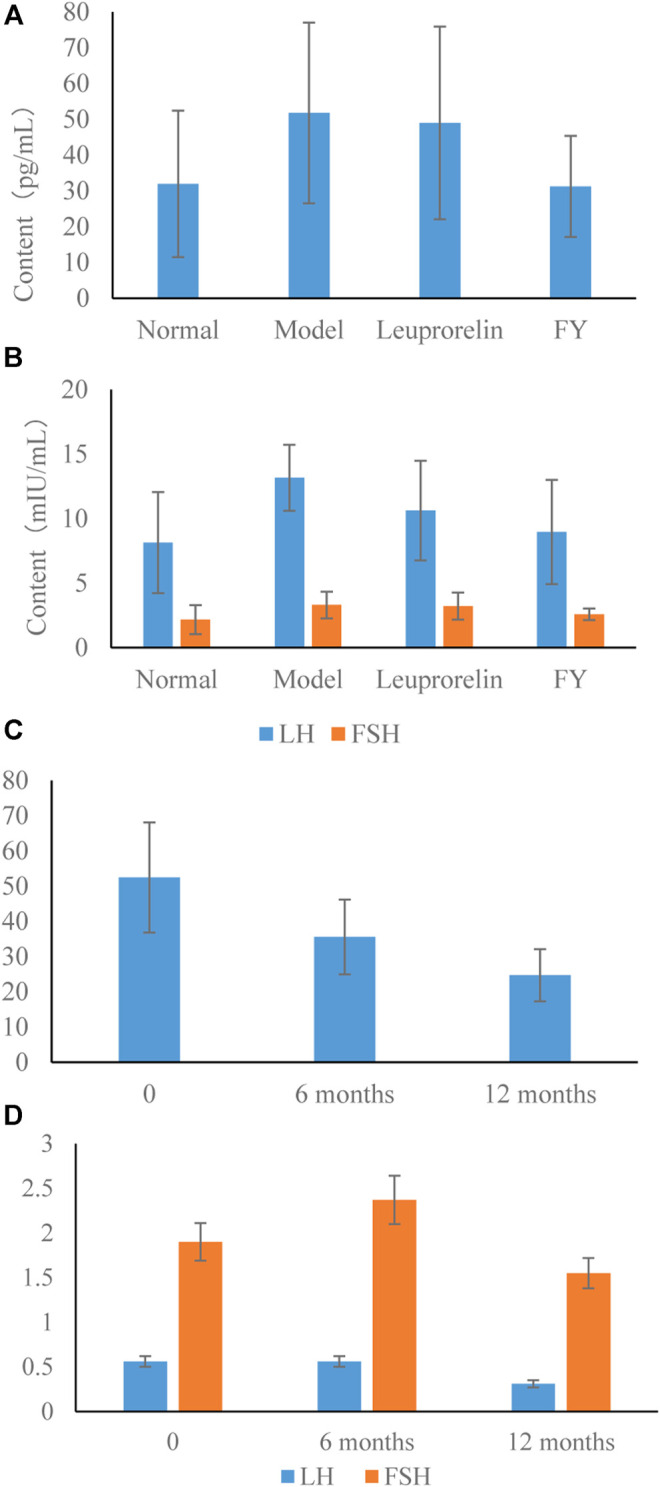
Effects of the FY on sex hormone levels in PP. **(A)** Serum estrogen (E_2_) levels among all groups of rats. **(B)** Serum luteinizing hormone (LH) and follicle-stimulating hormone (FSH) levels among all groups of rats. **(C)** Serum E_2_ levels among all groups of children. **(D)** Serum LH and FSH were detected among all groups of children.

Compared with the normal control group, E_2_, LH, and FSH levels in the model group were significantly increased (*p* < 0.01), indicating that the model of PP was successfully established. After treatment with the FY, the E_2_ and LH levels in rats with PP were significantly reduced compared with the model group (*p* < 0.01); and FSH levels were significantly reduced (*p* < 0.05), compared with the model group. However, in the leuprorelin group, only the LH levels showed a reduction (*p* < 0.05) (Figure 4A and Figure 4B).

### Retrospective Analysis of Cases

Retrospective analysis showed that 575 children who met the inclusion criteria were included. It showed that E_2_, LH, and FSH levels were significantly reduced after 12 months treatments (*p* < 0.01). Significant differences were noted in E_2_ levels between groups (*p* < 0.01). The FSH levels were significantly lower at 12 months after treatment compared with 6 months after treatment (*p* < 0.05) (Figures 4C,D).

## Discussion

The results of the analysis of the common targets of the FY revealed a total of 97 targets for Prunella vulgaris L. and *Carapax Trionycis*, which had common targets with other drugs. These findings suggest that these herbs had close synergistic effects with the other herbs. The following herbs: *Gentiana scabra Bunge, Chrysanthemum morifolium (Ramat.) Hemsl., Lycium chinense Mill, Alisma plantago-aquatica L, Scrophularia ningpoensis Hemsl, Paeonia suffruticosa Andrews* and *Rehmannia glutinosa (Gaertn.) DC* had a total of 51 targets. These findings indicate that these 7 herbs in the prescription also had synergistic effects. Furthermore, no common targets were detected for *Hordeum vulgare L, Concha Etreae, and Thalluslaminariae,* indicating that these 3 herbs are not key active ingredients in the formulation. Our previous study about data mining showed that the most frequently used herbs were *Anemarrhena asphodeloides Bunge*, *Rehmannia glutinosa (Gaertn.) DC*, *Phellodendron chinense C.K.Schneid*, *Paeonia suffruticosa Andrews*, and *Prunella Vulgaris L*. Common medicinal were cold and bitter, mostly attributed to the liver and kidney. The core of the herbs based on Zhibai Dihuangwan, and there were 4 kinds of herbs with FY.

At present, GnRHa is recommended to treat CPP, but not incompleteness precocious puberty in the relevant guidelines. The premature thelarche is the most common type of incomplete precocious puberty, 14–23% of which will develop to CPP (Zhu et al., 2008). So intervene as early as possible is the present clinical needs to solve the problem. Literature shows that Zhibai Dihuang Pill and Dabuyin Pill can treat precocious puberty (Liu and Wang, 2018; Wang and Zhao, 2019; Wang et al., 2020), but there is no indication for this in their instructions, so it belongs to off-label drug use.

The above-mentioned number of common targets of each herb is consistent with compatibility principles of the formulation. We identified main chemical constituents of FY using HPLC-MS/MS and confirmed that the main constituents related to the key targets in FY are Quercetin, kaempferol, Luteolin, Apigenin and Emodin. Analysis of the formulation reveals that the core components are mainly flavonoids, as well as kaempferol and quercetin, which are all phytoestrogens. Modern pharmacological studies have shown that phytoestrogens can make two-way adjustments, as they are similar to endogenous estrogen in structure and function. When the level of estrogen in the body is lower than the normal level, it can play an estrogen-like role, which can prevent and cure women's menopausal syndrome, prostate cancer, osteoporosis and cardiovascular diseases. On the other hand, when the level of estrogen in the body is higher than the normal level. such as breast hyperplasia, uterine fibroids and other diseases, it can produce estrogen antagonism and effectively weaken the response of target cells to estrogen (Chen et al., 2017; Cai and Zhanf, 2020).

Among the top 10 core targets selected, ESR1, IGF1 and other direct therapeutic targets are reportedly related to the onset and development of PP (Ye et al., 2011; Yang and Zhao, 2013; Wang et al., 2016), and are important targets in the treatment of PP. We further analyzed and clarified the core targets of biological function, gene function, and signal pathways. The results showed that the key components of the FY alone or combined were associated with transcription factor binding, transcription factor regulation, biological and cellular processes, such as GO or biological process-related gene/protein molecular function, and the estrogen signaling pathway, MAPK signaling pathway, and PI3K-Akt signaling pathways. These pathways mediate hormones that act on target tissues to achieve endocrine regulation. The E_2_ hormone binds with ESR1 to form a hormone-receptor complex that activates the estrogen signaling pathway. The MAPK signaling pathway and PI3K-Akt signaling pathway regulate the secretion of GnRH, LH, and FSH, as well as metabolic processes associated with bones. In addition, the growth hormone-insulin-like growth factor-1 (GH-IGF1) is the most important neuroendocrine factor associated with growth and development. Excessive IGF1 levels can inhibit GH secretion, and thereby inhibiting the growth of articular cartilage and epiphyseal cartilage, and retard growth in children (Su et al., 2017).

Treatment with GnRH analogues, such as Leuproline, which act by downregulating pituitary GnRH receptors (Carel et al., 2009), represent the standard of care for the treatment of CPP. The integrated pharmacological results suggest that the mechanism of the FY in the treatment of PP may include: competitive binding of E_2_ with ESR1and reduction in serum IGF1 concentrations. At the same time, animal experiments showed that the FY could reduce E_2_, LH, and FSH levels in rats with PP. Retrospective analysis of the medical records also showed that the FY could control the early symptoms of PP, and effectively inhibit E_2_ levels. The results of the animal experiments and retrospective analysis of medical records have confirmed the feasibility of studies of the mechanism of action of Chinese herbal compounds by integrative pharmacological methods.

In addition, the complex key targets of the FY and predictions based on the KEGG pathway analysis results show that the treatment may have effects on ovarian steroidogenesis, prostate and breast cancer, and other signaling pathways. These results are consistent with our previous clinical studies, which have shown that the effects of complex mixtures in girls with PP and ovarian cysts may be a more favorable intervention. Mixtures such as the FY may diminish ovarian cysts, regulate character development, improve liver function and qi stagnation, alleviate yin deficiencies and heat symptoms, reduce levels of E_2_, and retard bone aging and maturation. The present findings also provide a novel basis for the clinical application, and further research and development of the FY.

However, this study has some limitations that are worth mentioning. First, the reliability of the effects of FY against PP depends on database, so biological verification is necessary to evaluate the reliability of bioinformatics analysis *in vitro, in vivo* and *in silico*. Secondly, quantitative analysis of the synergistic effect of the main compounds should be investigated in the future.

## Conclusion

In conclusion, we combined methods of big data discovery with biological validation to study the mechanism of actions of the FY in PP at the systemic level. We used the TCM-IP database for the treatment of PP and considered various components and molecular mechanisms in the preliminary analysis. We determined that the FY acts through multiple component interactions with targets. The mixture also exerts its effects through multiple pathways involved in the regulation of PP, and may thus play a role in treatment of the condition. Whether other pathways or mechanisms predicted in this network pharmacological approach also contribute to the beneficial effects of the FY requires further investigation.

## Data Availability

The original contributions presented in the study are included in the article/Supplementary Material, further inquiries can be directed to the corresponding author.

## References

[B1] Cai X.-Y. Zhanf Z.-J. (2020). Pharmacological Effects of Phytoestrogens and Research Progress of Related Traditional Chinese Medicine. Chin. Med. J. Res. Prac. 34 (2), 75–78. 10.13728/j.1673-6427.2020.02.015

[B2] Carel J.-C. Eugster E. A. Rogol A. Ghizzoni L. Palmert M. R. (2009). Consensus Statement on the Use of Gonadotropin-Releasing Hormone Analogs in Children. Pediatrics 123 (4), e752–e762. 10.1542/peds.2008-1783 19332438

[B3] Chen M. Zhao P.-W. Zhao D. Wu H.-B. (2017). Research Progress on Pharmacological Action of Phytoestrogens in Traditional Chinese Medicine. Jiangsu J. Traditional Chin. Med. 49 (4), 82–85. 10.3969/j.issn.1672-397X.2017.04.032

[B4] Chinese Society of Pediatric Endocrinology and Metabolism (CSPEM) (2015). Consensus on Diagnosis and Treatment of Central Precocious Puberty. Chin. J. Pediatr. 53 (6), 412–418. 10.3760/cma.j.issn.0578-1310.2015.06.004 26310550

[B5] Gfeller D. Grosdidier A. Wirth M. Daina A. Michielin O. Zoete V. (2014). SwissTargetPrediction: a Web Server for Target Prediction of Bioactive Small Molecules. Nucleic Acids Res. 42 (W1), W32–W38. 10.1093/nar/gku293 24792161 PMC4086140

[B6] Hamosh A. Scott A. F. Amberger J. S. Bocchini C. A. McKusick V. A. (2004). Online Mendelian Inheritance in Man (OMIM), a Knowledgebase of Human Genes and Genetic Disorders. Nucleic Acids Res. 33 (Suppl. l_1), D514–D517. 10.1093/nar/gki033 PMC539987 15608251

[B7] Huang L. Xie D. Yu Y. Liu H. Shi Y. Shi T. (2018). TCMID 2.0: a Comprehensive Resource for TCM. Nucleic Acids Res. 46 (D1), D1117–D1120. 10.1093/nar/gkx1028 29106634 PMC5753259

[B8] Janet P. Juan M. R. Josep S. Francesco R. Emilio G. Ferran S. (2020). The DisGeNET Knowledge Platform for Disease Genomics: 2019 Update. Nucleic Acids Res. 48 (D1), D845–D855. 10.1093/nar/gkz1021 31680165 PMC7145631

[B9] Liu H.-L. Liu J. Liu G.-Q. (2009). Clinical Research on 60 Cases of Girls with Central Precocious Puberty Treated with Fuyou Mixture. Beijing J. Traditional Chin. Med. 28 (8), 588–589. 10.16025/j.1674-1307.2009.08.035

[B10] Liu J.-P. Wang H. (2018). Clinical Effect and Safety of Zhibai Dihuang Pill Combined with Dabuyin Pill in the Sexual Precocity of Girls. Chin. J. Hum. Sex. 27 (1), 64–67. 10.3969/j.issn.1672-1993.2018.01.019

[B11] Morishita H. Takemoto M. Kondo H. Higuchi K. Aono T. (1993). Induction of True Precocious Puberty by Neonatal Treatment with Danazol in Female Rats. Neurosci. Lett. 157 (1), 33–36. 10.1016/0304-3940(93)90636-y 8233026

[B12] Pan Y.-C. Liu J. Liu H.-L. (2019). Clinical Observation on the Treatment of Girl Ovarian Cysts Complicated with Precocious Puberty with Traditional Chinese Medicine Fuyou Mixture. Beijing J. Traditional Chin. Med. 38 (7), 700–703. 10.16025/j.1674-1307.2019.07.021

[B13] Pathan M. Keerthikumar S. Ang C.-S. Gangoda L. Quek C. Y. J. Williamson N. A. (2015). FunRich: An Open Access Standalone Functional Enrichment and Interaction Network Analysis Tool. Proteomics 15 (15), 2597–2601. 10.1002/pmic.201400515 25921073

[B14] Ru J. Li P. Wang J. Zhou W. Li B. Huang C. (2014). TCMSP: a Database of Systems Pharmacology for Drug Discovery from Herbal Medicines. J. Cheminform 6 (1), 13. 10.1186/1758-2946-6-13 24735618 PMC4001360

[B15] Shannon P. Markiel A. Ozier O. Baliga N.-S. Wang J.-T. Ramage D. (2003). Cytoscape: a Software Environment for Integrated Models of Biomolecular Interaction Networks. Genome Res. 13 (11), 2498–2504. 10.1101/gr.1239303 14597658 PMC403769

[B16] Sherman B. T. Lempicki R. A. (2009). Systematic and Integrative Analysis of Large Gene Lists Using DAVID Bioinformatics Resources. Nat. Protoc. 4 (1), 44. 10.1038/nprot.2008.211 19131956

[B17] Su H.-R. Tang L.-L. Yuan R.-F. Chen X.-X. Wei Q.-S. Deng W.-M. (2017). A Kidney-tonifying Herbal Fufang Effects the Bone Mineral Density in Senile Osteoporosis Mice by GH/IGF-1 axis. J. Pract. Med. 33 (15), 2459–2464. 10.3969/j.issn.1006-5725.2017.15.009

[B18] Szklarczyk D. Santos A. von Mering C. Jensen L. J. Bork P. Kuhn M. (2016). STITCH 5: Augmenting Protein-Chemical Interaction Networks with Tissue and Affinity Data. Nucleic Acids Res. 44 (D1), D380–D384. 10.1093/nar/gkv1277 26590256 PMC4702904

[B19] Wang W.-H. Xiang N. Deng Y.-P. Tang J.-Q. Zhou Y.-N. Zhou G.-W. (2016). Effect of Nourishing Yin and Shugan Prescription on Hypothalamus-Pituitary-Gonadal axis and Body Mass in Female Central Precocious Rats. Chin. J. Integrated Traditional West. Med. Intensive Crit. Care 23 (1), 71–75. 10.3969/j.issn.1008-9691.2016.01.017

[B20] Wang X.-J. Li P.-O. Feng C.-Z. (2020). Ultrasound Evaluation of the Clinical Efficacy of Zhibai Dihuang Pill Combined with Dabuyin Pill in the Treatment of Central Precocious Puberty in Girls. J. PEDIATRICS TCM. 16 (3), 72–76. 10.16840/j.issn1673-4297.2020.03.21

[B21] Wang Y.-H. Zhao X. (2019). Effect of Zhibai Dihuang Pills Combined with Diphereline in the Treatment of Childhood Precocious Puberty and Their Effect on Serum Hormone Levels. Med. J. Wuhan Univ. 40 (3), 444–448. 10.14188/j.1671-8852.2018.0973

[B22] Wishart D. S. Feunang Y. D. Guo A. C. Lo E. J. Marcu A. Grant J. R. (2018). DrugBank 5.0: a Major Update to the DrugBank Database for 2018. Nucleic Acids Res. 46 (D1), D1074–D1082. 10.1093/nar/gkx1037 29126136 PMC5753335

[B23] Wu Y. Zhang F. Yang K. Fang S. Bu D. Li H. (2019). SymMap: an Integrative Database of Traditional Chinese Medicine Enhanced by Symptom Mapping. Nucleic Acids Res. 47 (D1), D1110–D1117. 10.1093/nar/gky1021 30380087 PMC6323958

[B24] Xu H.-Y. Zhang Y.-Q. Liu Z.-M. Chen T. Lv C.-Y. Tang S.-H. (2019). ETCM: an Encyclopaedia of Traditional Chinese Medicine. Nucleic Acids Res. 47 (D1), D976–D982. 10.1093/nar/gky987 30365030 PMC6323948

[B25] Yan X.-N. Tian G.-X. He H.-R. Liu X.-M. Zhang J. Lv J. (2019). CTD Database Architecture and Data Acquisition Query and Extraction Method. Chin. J. Evid. Based Cardiovasc. Med. 11 (8), 905–909. 10.3969/j.issn.1674-4055.2019.08.03

[B26] Yang L.-Z. Zhao M.-F. (2013). Effect of Zhizao Granules on Serum Levels of Growth Hormone and Insulin-like Growth Factor 1 in Rats with Early Sexual Maturation. J. Changchun Univ. Traditional Chin. Med. 29 (1), 15–17. 10.3969/j.issn.1007-4813.2013.01.007

[B27] Ye J. Han X.-M. Li Y.-Q. Yang L.-L. Wu Y.-M. (2011). Effect of Kangzao Granule on Sex Hormone Level in Female Precocious Rats. Lishizhen Med. Materia Med. Res. 22 (3), 618–619. 10.3969/j.issn.1008-0805.2011.03.045

[B28] Zhu S.-Y. Du M.-L. Lin A.-H. (2008). An Analysis of Risk Factors for Premature Thelarche Converting into Complete Central Precocious Puberty. Chin. J. Pract. Pediatr. 23 (3), 174–176. 10.3969/j.issn.1005-2224.2008.03.007

